# Supplementary material for: Silencing KRIT1 Partially Reverses the Effects of Disturbed Flow on the Endothelial Cell Transcriptome
Source: Int J Mol Sci. 2025 May 2;26(9):4340. doi: 10.3390/ijms26094340 (PMC12072803; doi:10.3390/ijms26094340)
Supplement: Supplementary file 1 [file ijms-26-04340-s001.zip › ijms-3580666-supplementary.pdf]

Silencing *KRIT1* Partially Reverses the Effects of Disturbed Flow on the Endothelial Cell Transcriptome

Authors: Amelia Meecham[1,†], Sara McCurdy[1,†], Eduardo Frias-Anaya[1], Wenqing Li[1], Helios Gallego-Gutierrez[1], Phu Ngyuen[2], Yi-Shuan Li[2], Shu Chien[2], John Y.-J. Shyy[2], Mark H. Ginsberg[1,*], Miguel A. Lopez-Ramirez[*,1,3].

Author Affiliations: [1]Department of Medicine, [2]Department of Bioengineering, [3]Department of Pharmacology, University of California, San Diego, La Jolla, CA 92093

Running title: Silencing endothelial cell *KRIT1* under disturbed flow.

†, Authors contributed equally
*Corresponding Authors

Correspondence should be addressed to Miguel A. Lopez-Ramirez or Mark H. Ginsberg

9500 Gilman Dr., BSB 5096, La Jolla, CA 92093, Telephone: 858-534-4425

malopezramirez@health.ucsd.edu or mhginsberg@ucsd.edu

The authors declare no conflict of interest.

Supplemental

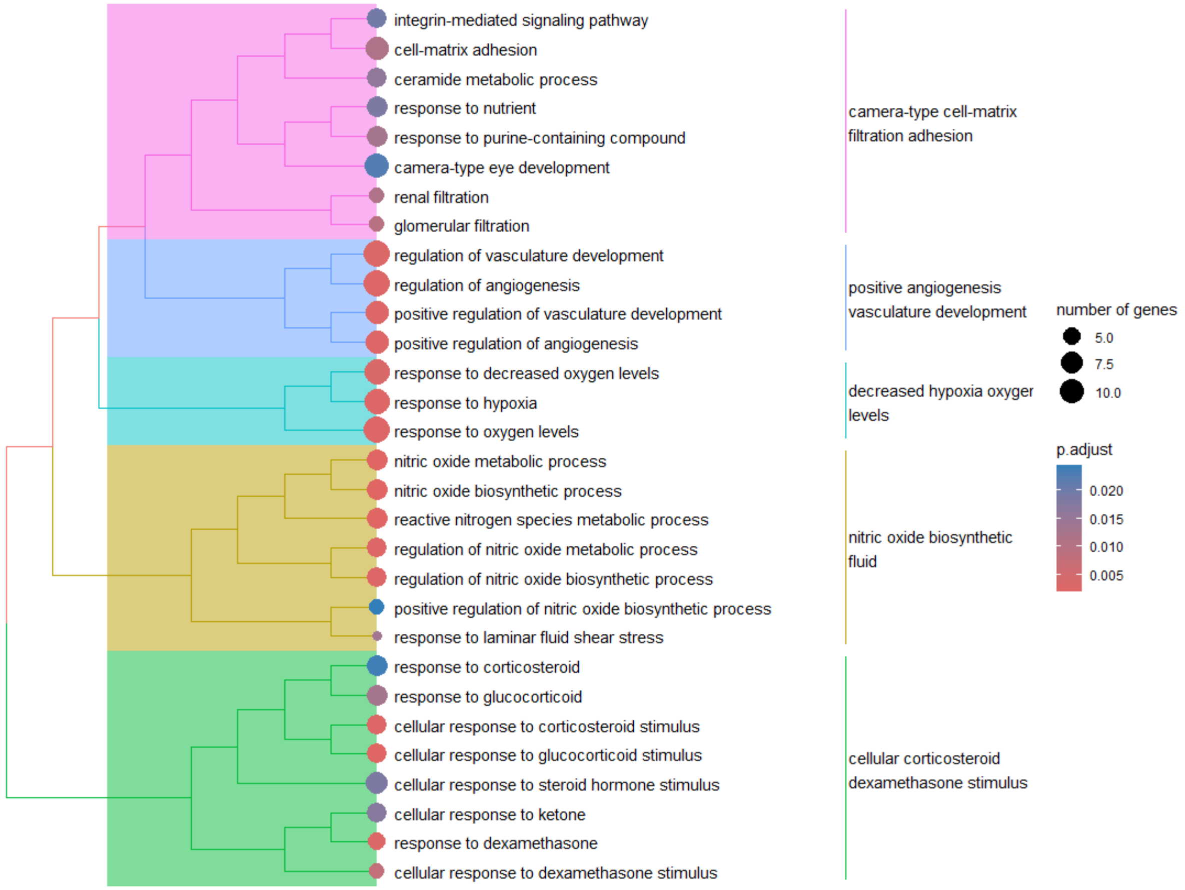

Supplemental Figure 1. Integration of significantly enriched pathways. Visualisation of pathway enrichment analysis. Tree plot clustering significantly enriched pathways using ‘clusterprofiler’. Genes overlapping in different pathways are adjusted for revealing 5 distinct branches used as the basis for heatmaps in Figure 2B.

Supplemental Table 1. Primer Sequences

Primer	Sequence
huKrit1 Fwd	CCA TCG TAC CTG TTA CCA AAC T
huKrit1 Rev	ACT GAC ACC TTC ACT TGT ACT G
hu KLF2 Fwd	AGA CCA CGA TCC TCC TTG A
hu KLF2 Rev	TCA CAA GCC TCG ATC CTC TA
hu KLF4 Fwd	GGT CTG TGA CTG GAT CTT CTA TC
hu KLF4 Rev	ACC CTG ATA TCC ACA ACT TCC
hu eNOS Fwd	CAT CAC CAG GAA GAA GAC CTT TA
hu eNOS Rev	TAC AGG ATT GTC GCC TTC AC

Supplemental Table 2. Differential expression analysis of key genes, those associated with inflammation and endothelial cell activation.

	Scr Os vs PS		Krit1 OS vs PS		Scr OS vs Krit1 OS		Scr PS vs Krit1 PS		Scr Stat vs Krit1 Stat	
	P VALUE	LOG2FOLD CHANGE	P VALUE	LOG2FOLDC HANGE	P VALUE	LOG2FOLDC HANGE	P VALUE	LOG2FOLD CHANGE	P VALUE	LOG2FOLD CHANGE
**KRIT1**	0.891	0.084	0.002	-1.545	<0.001	-1.534	<0.001	-1.461	<0.001	-1.781
**NOS3**	<0.001	1.247	0.405	-0.399	<0.001	1.551	<0.001	0.848	<0.001	1.240
**KLF2**	<0.001	4.768	<0.001	-3.761	<0.001	4.403	0.030	1.007	<0.001	4.756
**KLF4**	<0.001	3.612	0.011	-1.924	<0.001	3.871	<0.001	1.688	<0.001	5.140
**MCP1**	0.485	-0.733	0.959	-0.213	0.407	-0.751	0.260	-0.946	<0.001	-3.529
**VCAM1**	<0.001	0.847	0.434	-0.278	<0.001	1.081	<0.001	0.569	<0.001	0.803
**ICAM1**	0.163	1.079	0.564	-1.098	0.006	1.742	0.993	-0.018	0.006	-1.725
**SELE**	0.400	-0.876	0.794	-0.715	0.050	-1.539	0.041	-1.591	<0.001	-5.659
**SELP**	0.897	0.196	0.100	-2.417	0.238	-0.912	0.001	-2.221	0.187	0.981
**CXCL8**	0.323	-1.482	0.973	0.265	0.185	-1.694	0.384	-1.218	<0.001	-4.747
**IL6**	0.707	-0.460	0.920	-0.341	0.438	-0.706	0.356	-0.802	0.002	-2.085
